# A ten year review of the sickle cell program in Muhimbili National Hospital, Tanzania

**DOI:** 10.1186/s12878-018-0125-0

**Published:** 2018-11-14

**Authors:** Julie Makani, Furahini Tluway, Abel Makubi, Deogratius Soka, Siana Nkya, Raphael Sangeda, Josephine Mgaya, Stella Rwezaula, Fenella J. Kirkham, Christina Kindole, Elisha Osati, Elineema Meda, Robert W. Snow, Charles R. Newton, David Roberts, Muhsin Aboud, Swee Lay Thein, Sharon E. Cox, Lucio Luzzatto, Bruno P. Mmbando

**Affiliations:** 1 Muhimbili University of Health and Allied Sciences, Dar-es-Salaam, Tanzania; 2 University of Oxford, Oxford, UK; 3 Muhimbili National Hospital, Dar-es-Salaam, Tanzania; 4 Dar-es-Salaam University College of Education, Dar-es-Salaam, Tanzania; 5 University College London, London, UK; 6 Centre for Geographic Medicine Research, Kenya Medical Research Institute, Kilifi, Kenya; 7 National Institutes of Health, Bethesda, USA; 8 London School of Hygiene & Tropical Medicine, London, UK; 9 National Institute for Medical Research Tanga Centre, Tanga, Tanzania

**Keywords:** Sickle cell anemia, Africa, Tanzania

## Abstract

**Background:**

Africa has the highest burden of Sickle cell disease (SCD) but there are few large, systematic studies providing reliable descriptions of the disease spectrum. Tanzania, with 11,000 SCD births annually, established the Muhimbili Sickle Cell program aiming to improve understanding of SCD in Africa. We report the profile of SCD seen in the first 10 years at Muhimbili National Hospital (MNH).

**Methods:**

Individuals seen at MNH known or suspected to have SCD were enrolled at clinic and laboratory testing for SCD, haematological and biochemical analyses done. Ethnicity was self-reported. Clinical and laboratory features of SCD were documented. Comparison was made with non-SCD population as well as within 3 different age groups (< 5, 5–17 and ≥ 18 years) within the SCD population.

**Results:**

From 2004 to 2013, 6397 individuals, 3751 (58.6%) SCD patients, were enrolled, the majority (47.4%) in age group 5–17 years. There was variation in the geographical distribution of SCD. Individuals with SCD compared to non-SCD, had significantly lower blood pressure and peripheral oxygen saturation (SpO_2_). SCD patients had higher prevalence of severe anemia, jaundice and desaturation (SpO_2_ < 95%) as well as higher levels of reticulocytes, white blood cells, platelets and fetal hemoglobin. The main causes of hospitalization for SCD within a 12-month period preceding enrolment were pain (adults), and fever and severe anemia (children). When clinical and laboratory features were compared in SCD within 3 age groups, there was a progressive decrease in the prevalence of splenic enlargement and an increase in prevalence of jaundice. Furthermore, there were significant differences with monotonic trends across age groups in SpO2, hematological and biochemical parameters.

**Conclusion:**

This report confirms that the wide spectrum of clinical expression of SCD observed elsewhere is also present in Tanzania, with non-uniform geographical distribution across the country. Age-specific analysis is consistent with different disease-patterns across the lifespan.

## Background

The highest burden of sickle cell disease (SCD) is in Africa where up to 75% of the 300,000 global births of SCD per year occur [1] and where childhood mortality remains high, ranging between 50 and 90% [2]. Much of what is known about the spectrum of SCD is from cohort studies in USA [3], Europe and Jamaica [4–6], with few studies carried out in Africa [7–10] although it is recognized that there is a need for detailed prospective, epidemiological studies [11]. Tanzania is amongst the 5 countries in the world with the highest estimated number of newborns with SCD a year [12] (Nigeria 85,000, Democratic Republic of Congo 42,000, India 38,000; Tanzania 11,000 and Uganda 10,000 [1, 11]). The World Health Organization (WHO) has formally classified SCD as a major public health problem [13] and Tanzania, like other African countries has included SCD as a priority disease condition in their strategy for Non-Communicable Diseases (NCD) [13, 14]. In 2004, Tanzania established the Muhimbili Sickle cell (MSC) program [15], integrating research and healthcare in a countrywide referral hospital for all disease conditions, the Muhimbili National Hospital (MNH). The main goal was to conduct research that would delineate the spectrum of disease, identify the main causes of morbidity and mortality and provide evidence-based knowledge that would improve healthcare through the introduction of locally-appropriate interventions and policies. Preliminary information on this population, particularly mortality rates [16], rates and risk factors for malaria [17, 18], bacterial infections [19] and other important clinical features [20–23] has been published. The MSC program has also provided a platform to conduct studies to describe genetic determinants of clinical disease [24], successfully conducting one of the first genome-wide association studies in SCD in Africa [25].

In this paper, we outline the profile of the cohort in the MSC program enrolled over the first 10 years. We then describe the clinical and laboratory features of the SCD population enrolled, as well as the geographical distribution, based on self-reported ethnicity. This program demonstrates the feasibility of conducting longitudinal, cohort studies in Africa and this baseline report is setting the stage for further detailed description of the course and spectrum of disease over time.

## Methods

### Study site

Tanzania has a population of 45 million and is classified as a low income country. The MSC program was established through collaboration between Muhimbili University of Health and Allied Sciences (MUHAS), the oldest and largest biomedical university in Tanzania and MNH, the national referral hospital which is in Dar-es-Salaam on the Eastern coast.

### Study population

Individuals attending MNH who were known or suspected clinically to have SCD or related to SCD individuals were invited to the clinic for enrolment.

### Clinic procedures

The hospital had two clinics a week for SCD patients. During the course of the program, a third clinic was started. Patients were encouraged to visit the clinic in the event of an acute illness. All individuals who were enrolled were given a unique identifier number and hospital case number. Case report forms (CRF) were completed including demographic, clinical history and physical examinations. Residence information, including telephone number and consent to contact the family in case the patients did not attend clinic for more than 9 months were obtained. Management of SCD followed hospital guidelines which included prescription of folic acid 5 mg/day, investigations to identify underlying additional causes of anaemia, blood transfusion (BT) for those with haemoglobin< 5 g/dL, or heart failure. Individuals with splenomegaly requiring BT (> 3 times a year) were referred for splenectomy. Oral iron was prescribed to those with iron deficiency, defined by low serum ferritin or empirically to those with low MCV (< 80 fl) and MCHC (< 25 g/dL). Use of insecticide treated nets was emphasized to prevent malaria infections and chloroquine prophylaxis was prescribed until its use was discontinued due to high resistance. From 2010, SCD children under 5 years of age were prescribed daily oral penicillin.

### Laboratory procedures

At enrolment visits, peripheral blood samples in EDTA were analyzed for full blood count (haemoglobin (Hb), red blood cell count (RBC), mean corpuscular volume (MCV), mean corpuscular haemoglobin (MCH), mean corpuscular haemoglobin Concentration (MCHC)], white blood cell (WBC) and platelet count (PLT) (Pentra 60, Horiba ABX, Kyoto, Japan; Sysmex XT2000i, Hyogo, Japan). Nucleated RBC could not be differentiated from neutrophils by the haematology analyzer. Reticulocyte count (New methylene blue method; Sysmex XT2000i, Hyogo, Japan) and fetal haemoglobin (HbF) levels by High performance Liquid Chromatography (HPLC) (BioRad, Hercules, CA, USA) were obtained. SCD diagnosis was made by HPLC and Haemoglobin electrophoresis (Helena, Sunderland, Tyne & Wear, UK). Bilirubin total (BIL-T), bilirubin direct (BIL-D)], lactate dehydrogenase (LDH), alkaline phosphatase (ALP) and creatinine were assayed using a chemistry analyzer (Roche Cobas Mira, New York, USA or Abbott Architect, New York, USA). Daytime peripheral oxygen saturation (SpO_2_) was determined using a pulse oximeter (pulse oximetry; Nellcor, Pleasanton, CA, USA). Peripheral Oxygen desaturation was defined as < 95%.

### Data management and analysis

Data were managed using MySQL database (Sun Microsystems Inc., Santa Clara, California, USA). Analysis was performed using STATA and R statistical software (http://www.R-project.org/). Continuous variables with normal distribution were compared using t-test or ANOVA test and skewed continuous variables were compared using Wilcoxon sign rank or Kruskal Wallis tests, while categorical data were compared using chi-square tests. In order to evaluate the trend of different laboratory parameters, the mean difference between non-SCD and SCD were compared across the age groups. Some mean differences were log transformed to improve visibility of parameters with high and low levels of the difference. Weighted logistic regression was used to determine association between binary response variable and explanatory variables (clinical and haematological factors) accounting for under representation of non-SCD individuals. A *p*-value < 0.05 was considered to be statistically significant.

### Description of the MSC cohort

The description included the age structure and pattern of enrollment. SCD diagnoses were classified into 2 categories: SCD was for SS and non-SCD for those with AS and AA. SCD were almost all *β*^*S*^*/β*^*S*^ with a small fraction of S/β^0^ thalassaemia *(*estimated at 4% - unpublished data) and very rare *β*^*S*^/HPFH. SC or CC disease was not encountered. The age of patients was calculated at date of visit from the date of birth and three age group categories were defined: < 5, 5–17 and ≥ 18 (adults) years. Description of clinical and laboratory features between SCD and non-SCD individuals was limited to individuals in age group 5–17 years for the following reasons; 1) < 5 years age group is subject to considerable physiological changes; 2) ≥18 years age group is a heterogeneous group and it is not clear whether it consists of individuals with mild disease who have survived the early mortality of severe disease but who are symptomatic and therefore attend MNH for healthcare. The ethnic group of an individual was from self-reported ethnicity which is patrilineal. If this information was missing, the ethnic group of the mother was used. We plotted the geographical distribution of SCD in Tanzania based on the ethnicity of the SCD cases registered at MNH. The region where a particular ethnic group resides was used as a surrogate for the geographical distribution of SCD. Geographical coordinates of Tanzania and regional administrative boundaries were obtained from Global administrative boundaries website [26].

## Results

During the 10-year period (2004–2013), 6397 individuals were enrolled, with 3751 (58.6%) having SCD (> 95% *β*^*S*^*/ β*^*S*^). 3175 (49.6%) were male and 2393 (37.4%) were children aged < 5 years (Table 1). The median age at testing was higher in non-SCD than in SCD individuals (*p* < 0.001), while the sex ratio was similar between the two groups and there was no difference among those who were coming outside Dar (proxy for distance) compared to those coming from outside Dar es Salaam (Table 1). As the MSC program continued there was a statistically significant (*p* < 0.001) decrease in age of SCD individuals enrolled; the median age decreased from 9.1 years [Interquartile range (IQR): 5.1–14.1] in the first two years (2004–5) to 5.1 years (IQR: 2.3–11.1) during the last two years (2012–3).

**Table 1:** Characteristics of individuals enrolled in the Muhimbili Sickle Cell Programme in Dar-es-Salaam, Tanzania

Variable	Total	Non-SCD	SCD	Test statistic (*p*-value)
Number screened, n (%)	6397	2646 (41.4)	3751 (58.6)	
Median age (IQR)		9.3 (2.7–24.1)	6.9 (3.3–12.8)	z = 8.7 (< 0.001)
Age group (%)				
0–4	2393 (37.4)	956 (36.1)	1437 (38.3)	χ^2^ = 346.7 (< 0.001)
5–17	2592 (40.5)	813 (30.7)	1779 (47.4)	
18+	1360 (21.3)	842 (31.9)	518 (13.8)	
Missing	52 (0.8)	35 (1.3)	17 (0.4)	
Sex (%)				
Male	3175 (49.6)	1294 (48.9)	1881 (50.1)	χ^2^ = 0.96 (0.328)
Female	3222 (50.4)	1352 (51.1)	1870 (49.9)	
Place of birth (%)				
Dar es Salaam	3807 (59.5)	1445 (54.6)	2362 (63.0)	χ^2^ = 41.36 (< 0.001)
Others	2478 (38.7)	1143 (43.2)	1335 (35.6)	
Missing	112 (1.8)	58 (2.2)	54 (1.4)	
Place living				
Dar es Salaam	4446 (69.5)	1628 (61.5)	2818 (75.13)	χ^2^ = 1.06 (0.304)
Others	834 (13.0)	321 (12.1)	513 (13.9)	
Missing	1117 (17.5)	697 (26.2)	420 (11.2)	

Test statistics excluded missing values

**Fig. 1:**
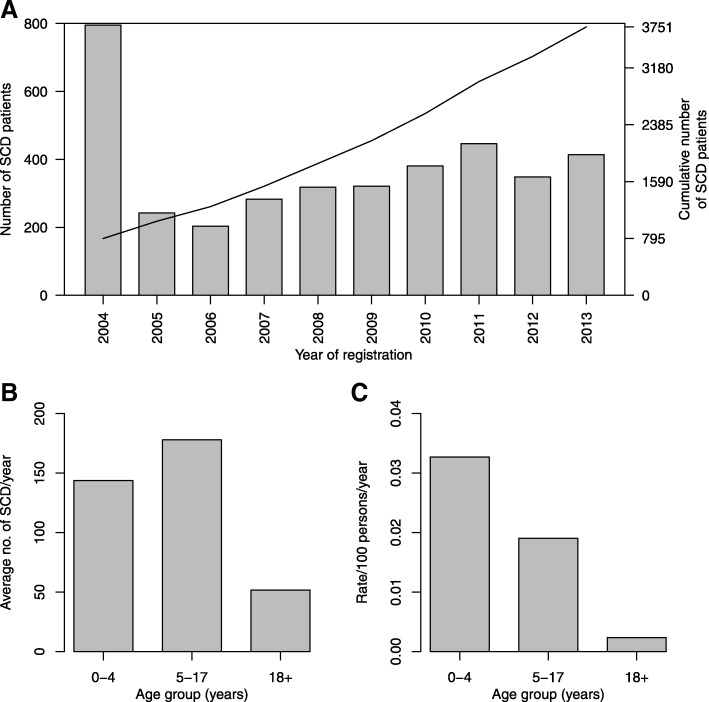
Pattern of enrollment of individuals with SCD over a 10-year period. Bars and line shows the annual enrolment and cumulative number of enrolment respectively (**a**). Average number of individuals enrolled per year by age group (**b**) and rate of enrolment per 100 persons per year by age group (**c**)

The trend of enrolment on annual basis for the SCD patients is shown in Fig. 1a. The largest number of individuals with SCD was enrolled, not surprisingly, during the first year of the program. Subsequently, the annual number enrolled was relatively steady, with the median number enrolled per month being 62 (IQR: 18.5–92). On average, the middle age group (5–17 years) was most abundantly represented (Fig. 1b). However, the rate of enrollment weighted for population size was highest amongst the youngest (< 5 years: Fig. 1c).

### Geographical distribution of SCD in Tanzania based on ethnicity of participant

**Fig. 2:**
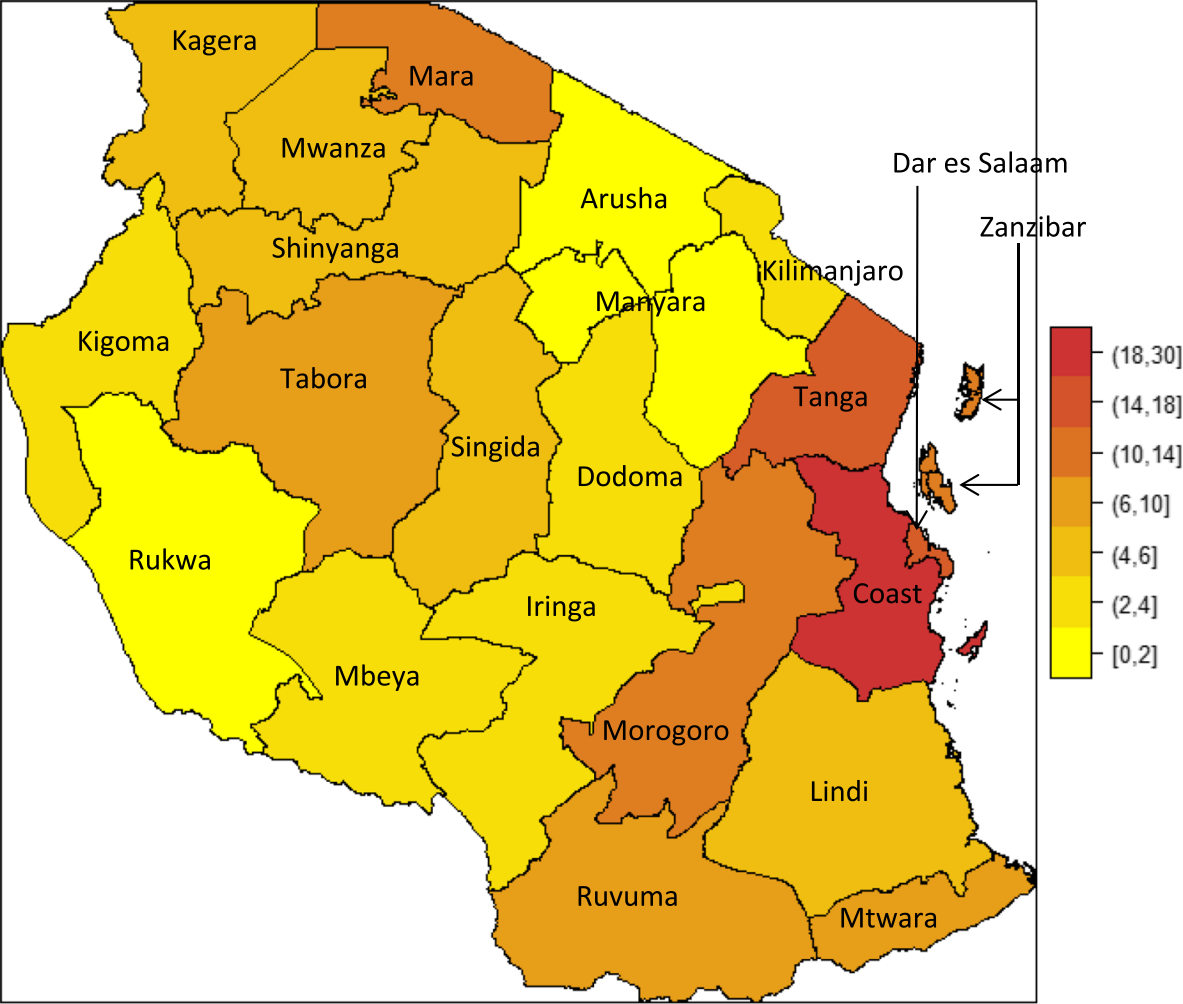
Geographical distribution of risk of sickle cell disease in Tanzania. Note: Prevalence is expressed (per 100,000) as the ratio of individuals with SCD enrolled in the MSC programme from a particular region to the total population of that region. Ethnicity is self-reported based on patrilineal tribe

Enrollment is a composite result of the variable prevalence of the Hb S gene, of migration patterns to Dar-es-Salaam and referral practices to MNH. With that proviso, we note that regions with the highest SCD cases were those along the coast (> 10 cases per 100,000 people), the North-Eastern part, which include regions of Coast, Tanga and Zanzibar and along the Lake Victoria zone (Tabora, Shinyanga, Kagera and Mara) with prevalence ranging from 5 to 12 cases per 100,000 people (Fig. 2). In contrast, Arusha, Manyara, Kilimanjaro and Rukwa regions had the lowest representation of SCD (< 2 cases per 100,000).

### Comparison of clinical and laboratory features of individuals (5 to 17 years) with and without SCD

**Fig. 3:**
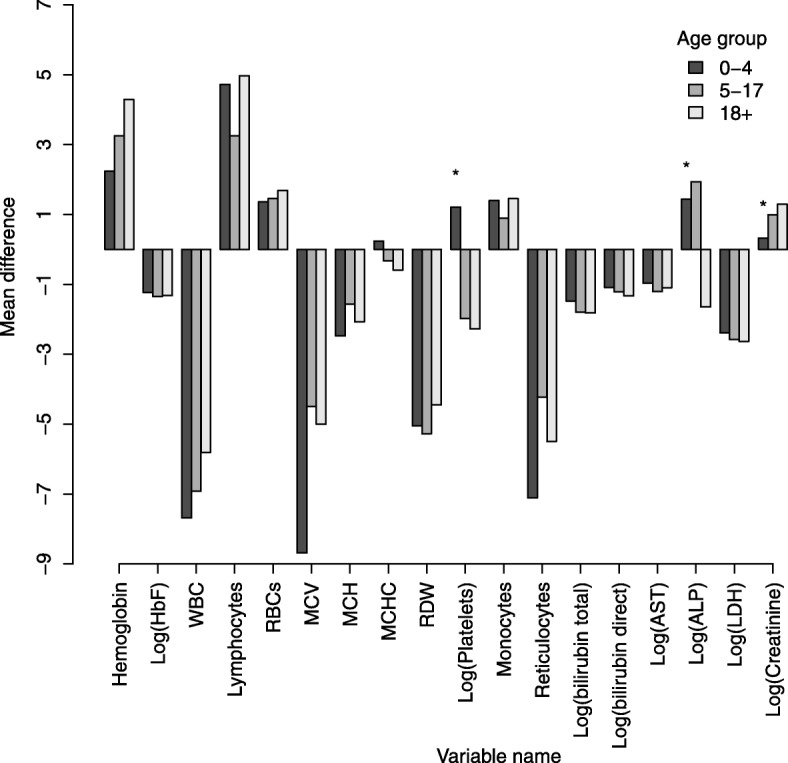
Distribution of mean difference of hematological parameters between non-SCD and SCD individuals by age-group. Only mean difference for platelets, ALP and creatinine for children < 5 years were not significant difference (*) between the non-SCD and SCD (*p* > 0.05)

Table 2 compares the clinical and laboratory parameters between non-SCD and SCD individuals aged 5–17 years for the reasons outlined under Methods section. Compared to non-SCD, the SCD group had statistically significant higher pulse rate and mean body temperature, but lower levels of blood pressure and peripheral oxygen saturation. The prevalence of low oxygen saturation (< 95%), jaundice and splenomegaly was significantly higher in SCD than in non-SCD group. Regarding laboratory features, SCD is a well-defined disease for which there is no *a priori* appropriate control group. Comparison of SCD and non-SCD individuals enrolled in the MSC programme, we find that all parameters were significantly different. The SCD group had a significantly lower Hb (g/dl) (7.4 vs 10.6), alkaline phosphatase and creatinine but higher levels of reticulocyte count, RDW, HbF, MCV, MCH, WBC, platelet count, total, direct and indirect bilirubin, aspartate transaminase, and lactate dehydrogenase. Similar trends for parameter levels were observed even when analysis was extended for all age groups, Fig. 3. The trends of mean difference were similar across the age groups, except for MCHC (non-SCD aged < 5 years had higher levels) and ALP (non-SCD aged > 18 years had lower levels) when compared to other groups. Only mean difference for platelets, ALP and creatinine for children < 5 years were not significantly different between the non-SCD and SCD groups (*p* > 0.05).

**Table 2:** Clinical and laboratory features of SCD and non-SCD individuals aged 5–17 years

	Non-SCD (HbAA, AS)		SCD (HbSS)		Test statistic (*p*-value)
	n	Estimate	n	Estimate	
Clinical features					
Age (years) (Median, IQR]	662	9.91 (7.1–13.1)	1010	9.5 (7.1–12.5)	χ^2^ = 5.37 (0.020)
Pulse rate (beats/min) [mean, 95%CI]	680	90.5 (89.2–91.8)	1585	94.0 (93.2–94.7)	*t* = − 4.79 (< 0.001)
Temperature(^o^c) [mean, 95%CI]	694	36.0 (35.8–36.4)	1628	36.5 (36.4–36.5)	*t* = − 3.63 (< 0.001)
Systolic Blood pressure (mm/Hg) [mean, 95%CI]	685	107.5 (105.8–109.2)	1462	104.9 (104.1–105.6)	*t* = 3.31 (0.001)
Diastolic blood pressure(mm/Hg) [mean, 95%CI]	684	67.5 (66.3–68.7)	1461	63.9 (63.3–64.5)	*t* = 5.96 (< 0.001)
Peripheral Oxygen saturation,(median, IQR)	662	99 (98,100)	1010	98 (96,100)	z = 11.78 (*p* < 0.001)
Peripheral Oxygen desaturation, [n/N (%)]		50/663 (7.5)		162/1009 (16.1)	χ^2^ = 26.2 (*p* < 0.001)
Jaundice, [n/N (%)]		45/309 (14.6)		943/1541 (61.2)	χ^2^ = 224.4 (*p* < 0.001)
Palpable Spleen, [n/N (%)]		25/451 (5.54)		201/1552 (12.9)	χ^2^ = 19.16 (< 0.001)
Laboratory features (95%CI)					
Hemoglobin (Hb) (g/dL)	759	10.6 (10.4–10.8)	1653	7.4 (7.3–7.4)	*t* = 32.34 (< 0.001)
Fetal hemoglobin (HbF)	422	1.7 (0.91–2.5)	803	6.8 (6.4–7.1)	*t* = −11.7 (< 0.001)
Mean Cell Volume (MCV) (fL)	762	75.2 (74.3–76.0)	1653	79.7 (79.1, 80.2)	*t* = −8.72 (< 0.001)
Mean Cell Hb (MCH) (pg)	743	24.5 (24.2–24.7)	1628	26.0 (25.8–26.2)	*t* = −9.12 (< 0.001)
Mean Cell Hb Concentration(MCHC) (g/dL)	760	32.2 (32.1–32.3)	1632	32.5 (32.4–32.6)	*t* = −4.00 (< 0.001)
Red Cell Distribution width (RDW) (%)	761	17.3 (17.0–17.6)	1636	22.6 (22.4–22.8)	*t* = −31.49 (< 0.001)
Reticulocyte (%)	294	7.1 (6.0–8.4)	583	11.2 (10.6–11.8)	*t* = −6.15 (< 0.001)
Absolute reticulocyte (×10^9^/L)	274	0.26 (0.21–0.31)	563	0.33 (0.32–0.36)	*t* = −2.42 (0.016)
White Blood Cells (WBC) (× 10^9^/L)	868	9.1 (8.6–9.5)	1582	15.6 (15.2–15.9)	*t* = − 23.5 (< 0.001)
Platelets (PLT) (× 10^9^/L)	763	349.3 (336.5–362.04)	1652	444.4 (434.5–454.3)	*t* = − 11.55 (< 0.001)
Bilirubin total (μmol/L)	271	10.3 (9.3–11.4)	930	59.7 (56.7–62.8)	*t* = −30.72 (< 0.001)
Bilirubin direct (μmol/L)	188	3.0 (2.5–3.6)	875	14.4 (13.6–15.2)	*t* = −19.08 (< 0.001)
Bilirubin indirect (μmol/L)	179	8.6 (7.4–9.9)	865	42.1 (39.5–44.8)	*t* = − 20.52 (< 0.001)
Aspartate AminoTransferase (AST) (U/L)	295	36.1 (32.6–39.5)	949	52.0 (49.7–54.5)	*t* = −7.69 (< 0.001)
Alkaline phosphatase (ALP) (IU/L)	294	353.6 (329.5–377.7)	953	267.5 (258.8–276.5)	*t* = 6.58 (< 0.001)
Lactate dehydrogenase (LDH) (U/L)	328	593.8 (548.0–639.5)	569	971.6 (932.0–1011.2)	*t* = − 12.28 (< 0.001)
Creatinine (μmol/L)	292	46.8 (44.7–48.9)	949	37.6 (36.6–38.5)	*t* = 8.36 (< 0.001)

### Clinical and laboratory features of SCD patients by age group

**Fig. 4:**
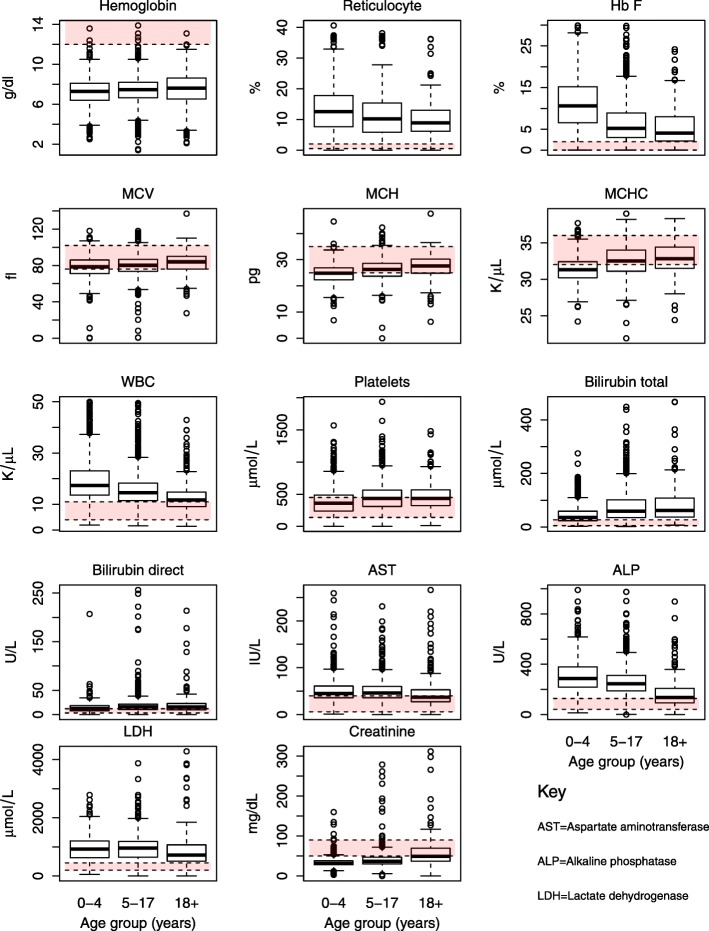
Distribution of selected laboratory parameters of SCD individuals by age-group

**Fig. 5:**
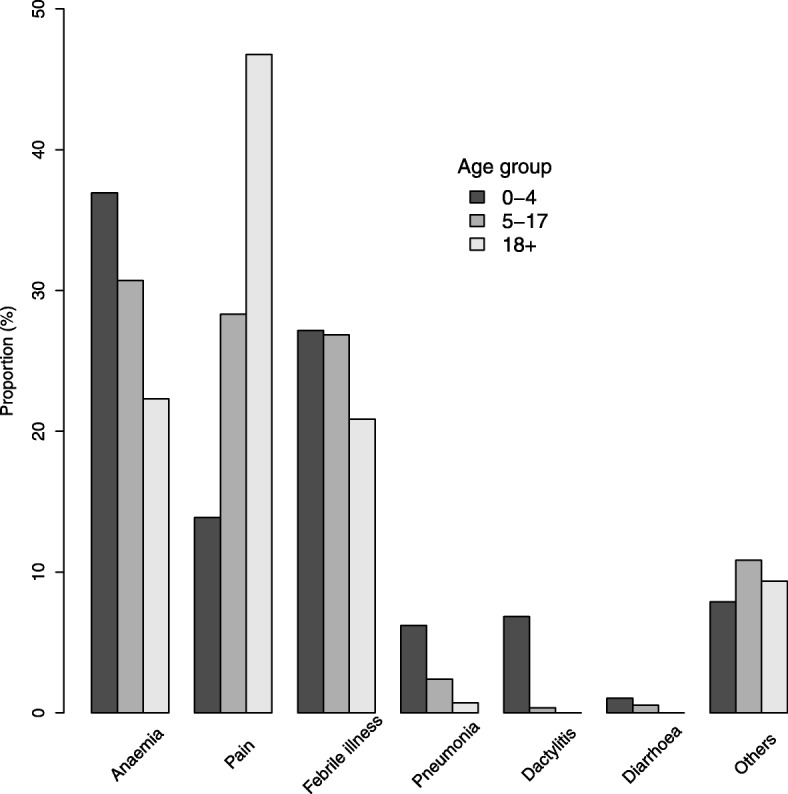
Conditions associated with hospital visits in the 12 months preceding enrolment visit

Evaluation of the clinical and laboratory features in three different age groups within the SCD population is presented in Table 3 and Fig. 4. There were statistically significant differences between the three age groups and interestingly, many of the parameters showed monotonic trends with increasing age. Notably, children aged 0–4 years had a significantly higher pulse rate and prevalence of palpable spleen, whilst peripheral oxygen saturation was lower in older SCD patients and prevalence of jaundice increased with age. The blood pressure (both systolic and diastolic) was significantly higher in the older SCD populations. For 1165 patients (31%), we obtained medical history on the causes of hospitalization in the 12 month period preceding enrollment (Fig. 5). Overall, the three commonest causes of hospitalization were pain, fever (including malaria) and severe anemia. Severe anaemia was the leading cause in children < 18 years (36.9% in 0–4 and 30.7 in 5–17 years), whereas pain was the main cause amongst adults aged ≥18 years (43.2%). Pain (12.9%), fever (12.0%) and malaria (11.2%) contributed equally in children below 5 years, while in children aged 5–17 years pain (27.4%) was the second contributing factor. Out of 469 patients who were admitted with symptoms of severe anaemia as the primary cause, severe anaemia (hb < 5 g.dL) was confirmed in 13.3% of the patients, 36 (7.7%) had pain, while only one patient had jaundice. Contrarily, of patients with Hb results (338) among those admitted with pain as the primary cause, only 11 (3.2%) had confirmed severe anaemia, while only three (out of 388) had jaundice.

**Table 3:** Clinical and laboratory features of SCD individuals in 3 age categories (< 5 years; 5–17 years and ≥ 18 years)

Variable	N	Age group (years)			Test statistic	*P*-value
		0–4 years	5–17 years	≥18 years		
Clinical features						
Age (years) (Median,IQR]	3265	2.5 (1·4–3.8)	9.5 (7.1–12.5)	23.7 (20.3–29.5)		
Pulse rate (beats/min) [mean, sd]	3238	110.11 ± 23.1	94.0 ± 15.1	83.5 ± 13.7	446.3^↓^	< 0.001
Temperature(^o^c) [mean, sd]	3413	36.5 ± 1.5	36.5 ± 1.0	36.3 ± 1.8	3.16^↓^	0.042
Systolic Blood pressure (mm/Hg) [mean, sd]	2993	97.9 ± 24.2	104.9 ± 14.4	116.6 ± 16.9	160.8^↑^	< 0.001
Diastolic blood pressure(mm/Hg) [mean, sd]	2993	58.8 ± 16.4	63.9 ± 11.5	70.7 ± 12.2	127.9^↑^	< 0.001
Peripheral Oxygen saturation,(median, IQR)	2713	99 (97–100)	98 (96–100)	98 (96–99)	13.7	0.001
Peripheral Oxygen desaturation, [n/N (%)]	2713	170 (15.4)	200 (16.4)	58 (14.9)	0.654	0.721
Jaundice, [n/N (%)]	3171	542 (45.0)	943 (61.2)	292 (68.4)	102.0^↑^	< 0.001
Pallor, [n/N (%)]	3102	447 (37.8)	573 (38.1)	142 (34.5)	2.06	0.357
Palpable Spleen, [n/N (%)]	3242	192 (15.5)	201 (12.9)	21 (4.7)	35.0^↓^	< 0.001
Laboratory features						
Hemoglobin (Hb) (g/dL)	3456	7.2 ± 1.4	7.4 ± 1.4	7.6 ± 2.1	10.24^↑^	< 0.001
Fetal hemoglobin (HbF)	1942	12.2 ± 7.9	6.8 ± 5.63	5.6 ± 4.7	182.9^↓^	< 0.001
Mean Cell Volume (MCV) (fL)	3454	78.1 ± 11.1	79.7 ± 11.7	82.4 ± 13.8	22.49^↑^	< 0.001
Mean Cell Hemoglobin (MCH) (pg)	3406	24.6 ± 3.6	26.0 ± 4.1	27.2 ± 5.0	84.11^↑^	< 0.001
Mean Cell Hemoglobin Concentration(MCHC) (g/dL)	3419	31.4 ± 1.7	32.5 ± 2.1	32.9 ± 2.1	169.58^↑^	< 0.001
Red Cell Distribution width (RDW) (%)	3440	23.8 ± 4.0	22.6 ± 3.8	21.1 ± 4.4	81.22^↓^	< 0.001
Reticulocyte (%)	1212	13.3 ± 7.8	11.3 ± 7.5	10.3 ± 7.0	14.45^↓^	< 0.001
Absolute reticulocyte (×10^9^/L)	1153	0.40 ± 0.35	0.33 ± 0.32	0.33 ± 0.62	5.06^↓^	0.006
White Blood Cells (WBC) (×10^9^/L))	3238	19.2 ± 8.3	15.6 ± 6.6	12.4 ± 5.2	170.95^↓^	< 0.001
Platelets (PLT) (×10^9^/L))	3453	377.6 ± 19.8	444.4 ± 205	450.7 ± 213.2	45.92^↑^	< 0.001
Log(Bilirubin total (μmol/L))	1706	3.62 ± 0.74	4.11 ± −.78	4.16 ± 0.81	76.3^↑^	< 0.001
Log(Bilirubin direct (μmol/L))	1561	2.45 ± 0.82	2.73 ± 0.80	2.79 ± 0.90	21.3^↑^	< 0.001
Log(Bilirubin indirect (μmol/L))	1545	3.28 ± 0.83	3.76 ± 0.92	3.80 ± 0.91	47.9^↑^	< 0.001
Aspartate Aminotransferase (AST) (U/L)	1750	53.2 ± 31.5	52.0 ± 38.3	48.2 ± 39.8	1.64	0.193
Alkaline phosphatase (ALP) (IU/L)	1758	314.2 ± 147.2	267.5 ± 140.5	179.2 ± 156.1	75.69^↓^	< 0.001
Lactate dehydrogenase (LDH) (U/L)	1196	962.2 ± 428.0	971.6 ± 481.1	918.4 ± 686.9	0.78^↑^	0.461
Log(Creatinine (μmol/L))	1744	3.49 ± 0.36	3.23 ± 0.39	3.94 ± 0.43	117.0^↑^	< 0.001

Variables (Hypoxia, Jaundice, Pallor, Palpable Spleen) were compared using χ^2^ test, while rest of variables were compared using F-test. ^↓^ Effect decrease with increase in age while ^↑^ indicate increase of the effect with age

The levels of Hb, MCV, MCH and MCHC showed a slight but significant upward trend, whereas RDW decreased with age. Reticulocyte counts were elevated in all age groups but they decreased with age. There were significant differences in the WBC counts with counts decreasing with age whereas platelet counts increased with age. Total, direct and indirect bilirubin all increased significantly with age (*p* < 0.001). AST and ALP decreased with age, however the decrease was only significant for ALP (*p* < 0.001).

## Discussion

This is the first study that provides a description of the spectrum of SCD in Africa from one of the largest single-centre SCD cohorts in the world. There has been relatively limited information about the spectrum of SCD in Africa when compared to information available from studies outside Africa. Most large series have been from the US, Jamaica and Europe which have involved study populations exceeding 2000 patients with prospective follow up for at least 10 years [4, 27, 28]. In Africa, there has been an increase in the number of similar studies, although the sample size has been smaller (500 to 1000 individuals) and duration has been shorter (up to 5 years) [7–9].

In the first two years of the MSC program, most of the patients enrolled were those already receiving clinical care at MNH, with the majority in the 5–17 year age group (Fig. 1b). This was therefore likely to be a survival cohort and highlighted two unmet needs: the urgency of newborn screening and of strengthening SCD pediatric care. The peak of ongoing enrollment was in 2011 that coincided with optimal number of personnel, weekly clinics and the doctor to patient ratio. This is also the period that the MSC program started working with the Ministry of Health of Tanzania with the aim of strengthening SCD services throughout the country, as part of the non-communicable diseases (NCD) program [29]. The age-specific rate of enrollment (Fig. 1c) shows a marked decrease with age: this suggests, unfortunately, a high rate of early mortality in the younger age group, which we have previously reported [16]. At the same time, it is important to note that 518 SCD individuals were aged ≥18 years, providing evidence of survival into adulthood and highlighting the need for adult services. It should be noted that the adult SCD population may comprise two distinct groups: (a) those with severe disease seeking healthcare due to acute and chronic complications, sometimes related to end-organ dysfunction; (b) those with relatively mild disease, who survived childhood.

We report heterogeneity in the geographical prevalence of SCD in Tanzania. The enrollment into the MSC program of patients from different parts of Tanzania will have been influenced by ease of access for residents in regions closer to MNH and/or by socio-economic status, whereby those with higher income are more likely to come to MNH. Nevertheless, it is interesting that the highest numbers were from the coastal regions, including Zanzibar, and from regions around Lake Victoria, despite the long distance between these regions and Dar-es-Salaam. These areas are known to have high malaria transmission^.^ On the other hand, there were lower numbers from regions close to Dar-es-Salaam such as Iringa, Manyara, Lindi, Kilimanjaro, where malaria transmission is less. It is noteworthy that, in spite of the ascertainment bias just mentioned, these data agree with previous estimates of β^S^ gene frequencies in Tanzania [1], although further research is needed to evaluate the genetic and environmental factors, other than malaria, that influence disease prevalence. Detailed micro-mapping of SCD prevalence will also aid planning of optimal health services, targeting areas with high prevalence.

In the initial analysis of features of SCD (Table 2) we compared SCD with non-SCD (HbAA and HbAS) individuals enrolled at the same time. The purpose of this comparison was not to illustrate the differences between SCD and non-SCD, as by definition SCD patients, who have life-long haemolytic anaemia would be discernably different from the non-SCD group. We anticipated that several parameters such as haemoglobin, reticulocytes and bilirubin levels would be higher. What we could not have predicted a priori was that the mean value of all parameters measured would be significantly different - even though, in many cases by a small measure (but, given the large numbers of individuals tested, in nearly all cases the *P*-value was less than 0.001). The SCD group when compared to non-SCD group had higher prevalence of jaundice and higher pulse rate. The blood pressure was lower in the SCD group, similar to previous reports of a low prevalence of hypertension in this SCD population as well as in the USA [23, 30]. The peripheral oxygen saturation was significantly lower in SCD: the difference was small, but in 16.1% of patients it was below 95%. This may be clinically signficant because it is related to the severity of anaemia [31] and to high 2,3-DPG levels, which increases deoxy-Hb S polymerization and sickling [32]. We and others have previously observed that low oxygen saturation is associated with increased cerebral blood flow velocity [33, 34], and higher risk of neurological complications [35, 36]. It was observed that there were low levels of red cell indices (MCV, MCH), which may be attributed in part to the high frequency of α-thalassaemia in this population (about 40% heterozygotes [37]): however, there is no a priori reason why this frequency should be any different in SCD versus non-SCD.

### Novel information

Most studies of SCD have focused on children and adults separately, reflecting the fact that most healthcare systems have dedicated but separate paediatric and adult services. The MSC program, instead, aimed to integrate the care of SCD patients across their life span. First, we noted that pain, infection and anaemia were the three most common causes of hospital visits (Fig. 5): but whereas in children the most common cause was anaemia, adults were more affected by painful episodes. This is similar to findings in other series, where 50% of adults experience painful episodes compared to only 10% in children [38]. Next, we analyzed by age groups all other data over the entire age span from infancy to adulthood. To our knowledge, this is one of the first studies in which such a systematic analysis has been attempted. Here we will focus only on a few observations which we think are important. First, mean level of Hb increased with age, though only slightly, and this was associated with a decrease in the mean reticulocyte count. This correlation is expected, as the increased reticulocyte count reflects a normal compensatory erythropoietic response to anaemia. The decreased reticulocyte count in older patients with SCD may also have reflected reduced erythropoietin levels in the context of the onset of chronic kidney disease, as evidenced by the higher creatinine in the older age group. An alternative explanation may possibly be a direct effect of vaso-occlusive events on the bone marrow resulting in reduced erythropoiesis with impaired reticulocyte production. Second, with respect to HbF, we expected that the mean HbF level would be highest in the youngest age group, since the physiological decrease in HbF from the level of about 80% at birth is much delayed in SS children – compared to controls – in at least the first 30 months of life [39]. However, the further decrease in HbF in the transition from the 5–17 age group to adulthood is somewhat paradoxical, because one would have expected that SCD patients with higher HbF would be, if anything, favored in terms of survival: since HbF was seen not to predict mortality in our previous work [33], this finding requires further research. Third, the mean values of red cell indices (MCV, MCH, MCHC) were lower in the younger age group which, in line with the fall in RDW with age, may mean that iron deficiency anemia is more common in the youngest patients (it is indeed common in Tanzania in the < 5 age group). Low MCV and MCH may also result from α-thalassaemia, which is generally regarded as an ameliorating factor in SCD; however, from these data and our previous work [33], low MCV does not seem to confer a survival advantage. Fourth, the mean WBC count decreased with age: which may suggest that the prevalence of infection is higher in children and decreases with age. However, the downward trend in WBC counts may also mean that the older age group is representative of less severe disease. In contrast, the mean platelet count increased with age, probably reflecting a decrease in splenic function: this explanation is supported by a lower prevalence of palpable spleen in the older age groups. The age-specific patterns in the level of WBC counts and platelets warrant further research, as the role of these blood components in the pathophysiology of SCD is being increasingly recognized [40]. Fifth, the mean level of bilirubin increases with age, in keeping with the observation that prevalence of jaundice increased with age. This may be explained by an increase in the rate of extravascular haemolysis; liver dysfunction is less likely since there was a decrease in the mean levels of ALP and AST (albeit not statistically significantly with respect to AST). We must also consider that the higher levels of ALP in the younger age groups could be related to increased bone tissue turnover associated with growth. A striking observation was the high level of LDH (more than 3 times the upper limit of normal) in all age groups, which suggests a significant level of intravascular haemolysis, although cardiac and liver dysfunction may also contribute. Further research is required to quantitate the age-specific rate of haemolysis and whether there is a change with age in the relative rates of intravascular versus extravascular haemolysis. Finally, the mean creatinine level increased with age, although most values remained within the normal range. The creatinine level on its own is not a sensitive index of early renal disease, but one wonders whether in individual cases the trend of creatinine with age may be a prelude to renal insufficiency, a known complication of SCD. It should be noted that the age-related trends in clinical and laboratory parameters in this report were also reported by Aliyu et al. [41] in Nigeria (208 individuals with SCD; Youngest 10 years; 7% > 35 years).

In one of the first systematic studies of SCD patients, Nagel and colleagues [42] noted that the β^*S*^ haplotype may account for the great variability in the clinical symptomatology and severity of SCD. In this population in Tanzania, this layer of heterogeneity is largely removed, since nearly all patients are homozygous for the Central African Republic (CAR) haplotype (previously referred to as Bantu haplotype) [43]: However, the spread of values remains just as great (Fig. 4); for example, the mean hematocrit level that Nagel et al. [42] reported for SS patients with CAR haplotype was 23.1, which corresponds almost to the mean Hb of 7.4 in the Tanzania population with the same haplotype, although the range in Tanzania is wider. Although the β-globin haplotype is regarded as a major determinant of HbF production, we found (Fig. 4) a markedly skewed distribution, whereby the top 2.5% of patients had HbF values some 4 times higher than the mean. Genomic studies conducted in SCD in Tanzania have already reported variability in prevalence of and co-existence of genomic loci that influence HbF, α-thalassemia, and G6PD levels [25, 37]. Further research is required, and is ongoing, to increase understanding in the complex interplay of genetic, environmental and socio-economic factors influencing variability of disease spectrum [44].

### Limitations

Because enrolment into the SCD cohort was hospital-based, the study may not have captured individuals with mild disease. The non-SCD group was not a population-based age- and sex-matched control group: rather, it was a ‘convenient’ comparison group consisting of patient family members and of individuals enrolled among those suspected to have SCD: hence they may have other medical conditions. Indeed, the clinical and laboratory features were often not normal. We did not separate controls with HbAA from those with HbAS. This is broadly justified because sickle cell trait subjects have no cells containing exclusively HbS and are haematologically normal. However it is possible that some parameters may differ in AA versus AS subjects: this requires further study. Finally, the work reported here is that from cross-sectional analysis of the enrolment cohort. Although the study was conducted over a 10 year period, we are not yet presenting longitudinal data.

## Conclusion

This work demonstrates that in an African country it is feasible to enrol, across all age groups, a large number of SCD patients and to assess in considerable detail their clinical and laboratory manifestations. Not surprisingly, in first approximation the clinical spectrum of SCD is similar to that observed elsewhere. However, the age distribution is different, unfortunately reflecting, at least in part, early mortality and delayed diagnosis due to the absence of newborn screening. The prevalence of severe anaemia is high, probably reflecting less blood transfusion treatment and other environmental factors, including malaria and a higher rate of bacterial infection.

SCD has been recognized as a major public health problem: as such, it was essential for us to establish not just its size, but also its spectrum. While this paper was in its first draft the first SCD patient successfully cured with gene therapy was reported [45] and the American Society of Hematology issued a *Call to Action on Sickle Cell Disease*. We feel strongly that in Africa the transition from taking stock to taking action is overdue: we must implement urgently beneficial measures such as newborn screening, infection prophylaxis, optimization of blood transfusion practice and the use of hydroxyurea; and we must also contribute to research on potentially curative treatments.

## Abbreviations

AA: Haemoglobin AA (normal haemoglobin)
ALP: Alkaline phosphatase
AS: Haemoglobin AS (sickle cell trait)
AST: Aspartate aminotransferase
BIL-D: Bilirubin direct
BIL-T: Bilirubin total
BT: Blood transfusion
CC: Haemoglobin CC
CRF: Case report forms
EDTA: Ethylenediaminetetraacetic acid
Hb: Haemoglobin
HbF: Fetal haemoglobin
HPFH: Hereditary persistence of fetal haemoglobin
HPLC: High performance Liquid Chromatography
IQR: Inter quartile range
LDH: Lactate dehydrogenase
MCH: Mean corpuscular haemoglobin
MCHC: Mean corpuscular haemoglobin Concentration
MCV: Mean corpuscular volume
MNH: Muhimbili National Hospital
MSC: Muhimbili Sickle cell
MUHAS: Muhimbili University of Health and Allied Sciences
NCD: Non-Communicable Diseases
PLT: Platelet count
RBC: Red blood cell count
RDW: Red cell distribution width
SC: Haemoglobin SC
SCD: Sickle cell disease
SS: Haemoglobin SS
UK: United Kingdom
USA: United States of America
WBC: White blood cell
WHO: World Health Organization
β^S^: Beta globin
